# Invariant heart beat span versus variant heart beat intervals and its application to fetal ECG extraction

**DOI:** 10.1186/1475-925X-13-163

**Published:** 2014-12-12

**Authors:** Huawen Yan, Hongxing Liu, Xiaolin Huang, Ying Zhao, Junfeng Si, Tiebing Liu

**Affiliations:** School of Electronic Science and Engineering, Nanjing University, Xianlin Campus, Nanjing, 210023 China; Nanjing General Hospital of Nanjing Military command, Nanjing, 210002 China

**Keywords:** Fetal ECG extraction, HRV, Template, Comb filter

## Abstract

**Background:**

The fundamental assumptions for various kinds of fetal electrocardiogram (fECG) extraction methods are not consistent with each other, which is a very important problem needed to be ascertained.

**Methods:**

Based on two public databases, the regularity on ECG wave durations for normal sinus rhythm is investigated statistically. Taking the ascertained regularity as an assumption, a new fECG extraction algorithm is proposed, called Partial R-R interval Resampling (PRR).

**Results:**

Both synthetic and real abdominal ECG signals are used to test the algorithm. The results indicate that the PRR algorithm has better performance over the whole R-R interval resampling based comb filtering method (RR) and linear template method (LP), which takes advantages of both LP and RR.

**Conclusions:**

The final drawn conclusion is: (1) the proposition should be true that the individual’s heart beat span is invariable for normal sinus rhythm; (2) the proposed PRR fetal ECG extraction algorithm can estimate the maternal ECG (mECG) more accurately and stably even in the condition of large HRV, finally resulting in better fetal ECG extraction.

## Background

Non-invasive fetal electrocardiogram (fECG) extraction is a classic dilemma in biomedical signal processing. Researchers put forward various kinds of methods to solve this problem. These methods can be classified into: (1) multi-channel methods, like principal component analysis and its variants [1–3], independent component analysis [4, 5], periodic component analysis (piCA) [6–9], some wavelet based methods [10, 11], adaptive method [12–14] and so on; (2) single-channel methods, such as singular value decomposition (SVD) [15], R-R interval resampling based comb filter (RR) [16], linear template methods (LP) [17–21] and so on; (3) and fusion methods [22–25]. A detailed description about these methods can be found in literature [26].

Among the above three kinds of methods, the single-channel methods are always used to subtract the maternal electrocardiogram (mECG) component from the mixed abdominal ECG (aECG) recording. Since mECG component usually takes up the highest proportion in aECG, the accurate estimation of mECG component is a key procedure to obtain final high-quality fECG.

When using SVD [15] and RR [16] methods to estimate mECG component, R-R interval resampling is required because of the periodicity and the heart rate variability (HRV) of mECG signal. In essence, an underlying hypothesis is taken that the lengths of various waves in each cardiac cycle are proportional to the length of the R-R interval (heart beat interval).

Linear template (LP) [17–21] methods are simple, effective and widely used mECG estimation methods. In LP methods, the wave complexes in current cardiac cycle are estimated by summing the weighted wave complexes from neighbour cardiac cycles, depending on at least the peak detection of each QRS complex for different heart cycles. In essence, it is assumed that when heart beat interval is time-variant, the heart beat span (i.e. the length from the beginning of P wave to the end of T wave) is stable. So no resampling step is taken in current LP methods.

For the above two assumptions, there is no clear conclusion on which one is correct. In [27, 28], it is said that the length from the beginning of P wave to the peak of R wave (Ps-R) is weakly dependent on heart rate, while in literatures [21, 29] the authors think it depends on the instantaneous heart rate. As for the length from the peak of R wave to the end of the T wave (R-Te), literatures [27, 28] have the opinion that the T wave is strongly dependent on heart rate, becoming narrower and closer to the QRS complex at rapid heart rates, while the opposite view is given in [21]. In [28], it is considered that the length from the end of T wave to the beginning of next P wave (Te-Ps) is strongly dependent on the heartbeat duration. So far, we have not seen literatures to ascertain such disagreement problem for normal sinus rhythm.

Obviously, the above disagreement problem is related to whether SVD, RR and LP methods are used correctly. It is a very crucial thing for designing a high-quality fECG extraction method, especially when the mECG has large HRV.

Based on MIT Normal Sinus Rhythm Database (NSRDB as Set A) [30] and the MIT-BIH Arrhythmia Database (ADB as Set B) [30, 31], the problem is investigated in this paper and a regularity comes out that heart beat span is invariant for variable heart beat intervals. Then we propose a new corresponding single channel fECG extraction algorithm based on the regularity. Both HRV and periodicity are considered in the method. And its effectiveness is verified by synthetic and real recordings.

## Methods

### Investigation on regularity of ECG waves duration

**Figure 1:**
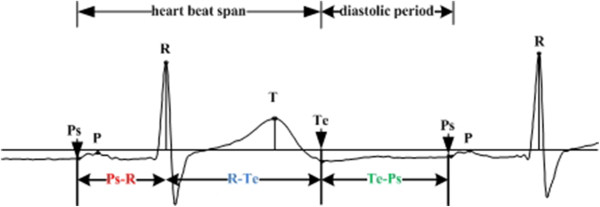
**A complete cardiac cycle and some feature points.**

A typical complete cardiac cycle is showed in Figure 1. Ps is the beginning point of P wave, and Te is the end point of T wave. Each cardiac cycle begins with a Ps point, including P wave, QRS complexes, T wave and then ends at the next Ps point. Let’s define the period from Ps to Te as heart beat span, and the period from Te to the next Ps as diastolic period.

The databases selected as research resources are Set A [30] and Set B [30, 31]. Set A is a normal sinus rhythm database, including 18 long-time records, with two channels for each record, and the sampling frequency is 128 Hz. And Set B is a database containing 48 two-channel recordings with different degree of arrhythmia; the sampling frequency is 360 Hz.

**Figure 2:**
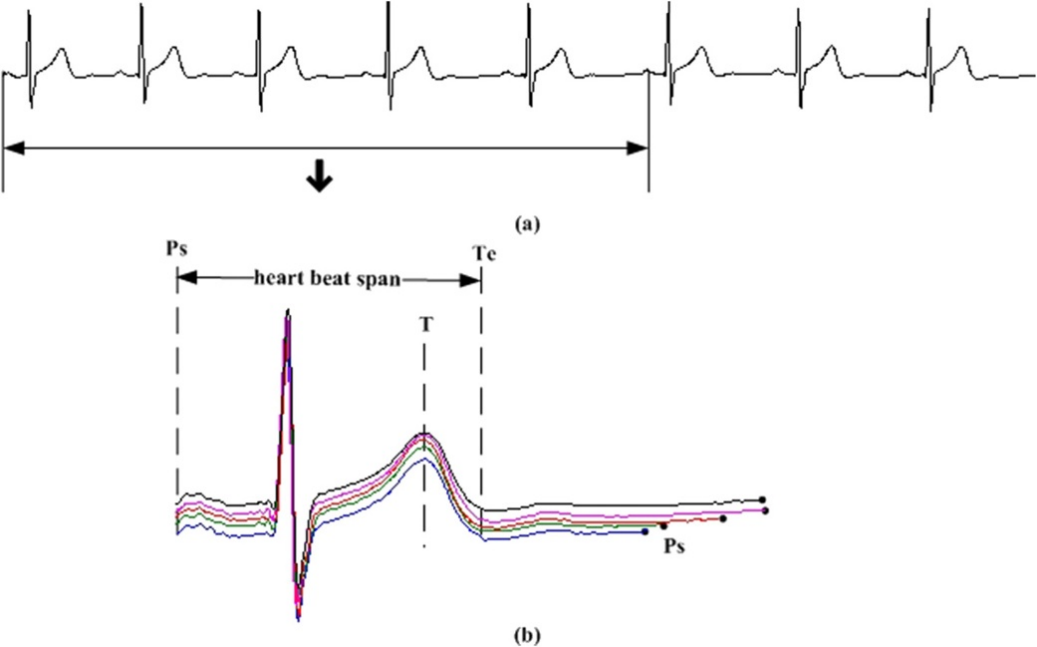
**Duration regularity for different cardiac cycles. (a)** data from the first channel of “19093”, **(b)** durations of five cardiac cycles.

**Figure 3:**
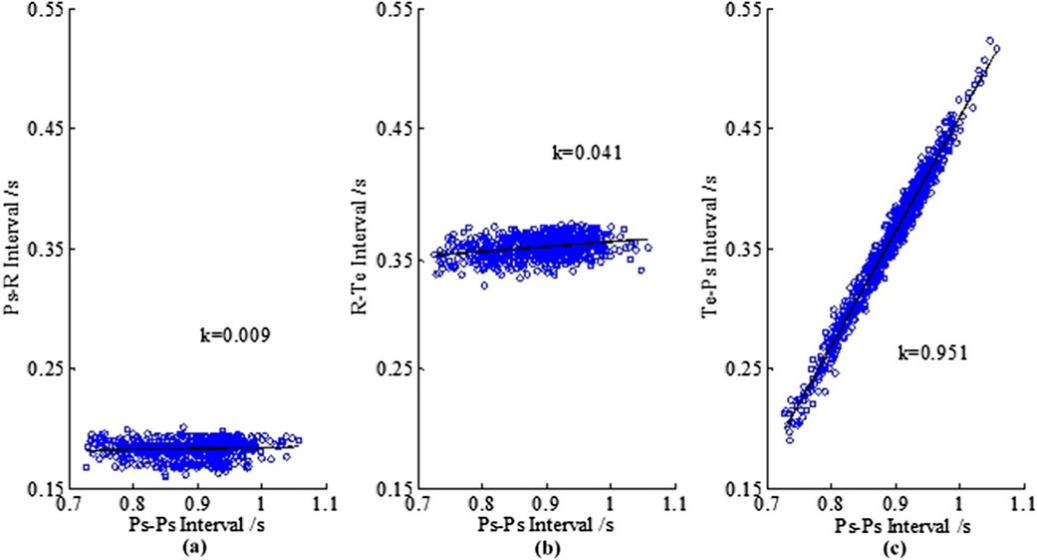
***t***_***Ps-Ps***_ **,** ***t***_***Ps-R***_ **,** ***t***_***R-Te***_ **,** ***t***_***Te-Ps***_ **and the gradients (k) (data: 19093). (a)** *t*_*Ps-R*_ vs. *t*_*Ps-Ps*_, **(b)** *t*_*R-Te*_ vs. *t*_*Ps-Ps*_, **(c)** *t*_*Te-Ps*_ vs. *t*_*Ps-Ps*_.

**Figure 4:**
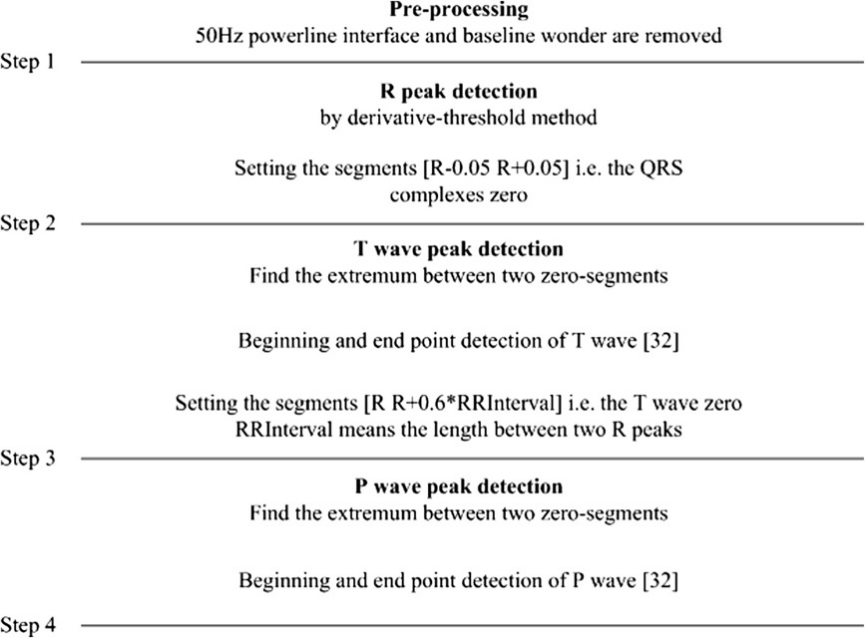
**The block diagram for characteristic wave detection of ECG signal** [32] **.**

Take the record “19093” from Set A as an example. A period from its first channel is given in Figure 2(a) and the first 5 cardiac cycles are depicted in Figure 2(b). It is quite obvious that the heart beat spans of the five cardiac cycles are almost the same, while the diastolic periods are quite different in length. The length of Ps-Ps (*t*_*Ps-Ps*_), the length of Ps-R (*t*_*Ps-R*_), the length of R-Te (*t*_*R-Te*_) and the length of Te-Ps (*t*_*Te-Ps*_) for the first 500 seconds-length data of record “19093” are illustrated in Figure 3. Details about how these characteristic waves are detected can be seen in Figure 4; an eye inspection is a must to ensure the validation of the detected results. And the final results are exported after manually correcting the inappropriate detections. Gradients (*k*s) for regression lines of *t*_*Ps-R*_ vs. *t*_*Ps-Ps*_, *t*_*R-Te*_ vs. *t*_*Ps-Ps*_ and *t*_*Te-Ps*_ vs. *t*_*Ps-Ps*_ are also calculated and listed in Figure 3. Since *k* for *t*_*Te-Ps*_ vs. *t*_*Ps-Ps*_ is nearly one and *k*s for the other two are almost zero, we can say that the length differences of Ps-Ps intervals are reflected totally by the change of *t*_*Te-Ps*_.

The above result on record “19093” presents that the *t*_*Ps-R*_ and *t*_*R-Te*_ are quite stable compared with *t*_*Ps-Ps*_. That is, there exists so called invariant heart beat span with the variant heart beat intervals. A further validation with the databases is given below.

First, because of the difficulty in detecting Ps and Te points accurately, for convenience we replace Ps-R by P-R interval, R-Te by R-T interval, Te-Ps by T-P interval and Ps-Ps by R-R interval for statistics’ validation. If the above conclusion from data “19093” is correct, the both substitute intervals P-R and R-T also should be stable, and T-P interval and R-R interval should always vary accordingly. Then, twelve records from Set A and seven from Set B with obvious P and T waves are selected. The first 500 seconds (s) data for each selected record are cut out for statistical analysis. Note that pieces that P or T wave is not clear are thrown away.

As in Table 1 shown, for the nineteen records the lengths of T-P (*t*_*T-P*_) and R-R interval (*t*_*R-R*_) change all the time, their standard deviation (std) are about 10^-2^; the lengths of P-R (*t*_*P-R*_) and R-T interval (*t*_*R-T*_) are comparatively stable, their std are 10^-3^. For *t*_*P-R*_ vs. *t*_*R-R*_ and *t*_*R-T*_ vs. *t*_*R-R*_ their gradients are in the order of 10^-2^; for *t*_*T-P*_ vs. *t*_*R-R*_ the gradients are bigger than 0.85. Considering the std and gradients, we think the results are consistent with that on record “19093”. The conclusion agrees with the underlying hypothesis taken by LP. It also illustrates the irrationality lying in SVD [15] and RR [16] methods.

**Table 1:** ***t***_***P-R***_ **,** ***t***_***R-T***_ **,** ***t***_***T-P***_ **and** ***t***_***R-R***_ **for the nineteen records in Set A and Set B**

Record no.	***t***_***P-R***_		***t***_***R-T***_		***t***_***T-P***_		***t***_***R-R***_	
	mean (s)	std	mean (s)	std	mean (s)	std	mean (s)	std
1	0.108	0.006	0.278	0.009	0.565	0.031	0.951	0.032
2	0.159	0.011	0.222	0.008	0.316	0.030	0.697	0.028
3	0.167	0.009	0.284	0.016	0.310	0.074	0.760	0.069
4	0.142	0.010	0.217	0.012	0.455	0.073	0.813	0.071
5	0.162	0.005	0.252	0.006	0.401	0.054	0.814	0.054
6	0.119	0.009	0.268	0.009	0.564	0.082	0.951	0.080
7	0.106	0.010	0.242	0.010	0.399	0.045	0.747	0.043
8	0.123	0.008	0.223	0.011	0.217	0.063	0.563	0.065
9	0.131	0.005	0.214	0.010	0.346	0.046	0.691	0.052
10	0.135	0.006	0.212	0.012	0.293	0.082	0.641	0.088
11	0.147	0.005	0.232	0.004	0.513	0.059	0.892	0.060
12	0.164	0.006	0.220	0.009	0.241	0.043	0.624	0.047
13	0.150	0.007	0.314	0.014	0.499	0.049	0.962	0.059
14	0.136	0.007	0.232	0.008	0.388	0.014	0.756	0.016
15	0.156	0.007	0.308	0.009	0.728	0.029	1.192	0.028
16	0.163	0.008	0.305	0.010	0.438	0.022	0.906	0.023
17	0.203	0.006	0.320	0.010	0.671	0.119	1.194	0.122
18	0.165	0.010	0.231	0.017	0.417	0.045	0.813	0.048
19	0.156	0.006	0.354	0.017	0.444	0.042	0.954	0.043

### Partial R-R interval resampling based fECG extraction Algorithm (PRR)

**Figure 5:**
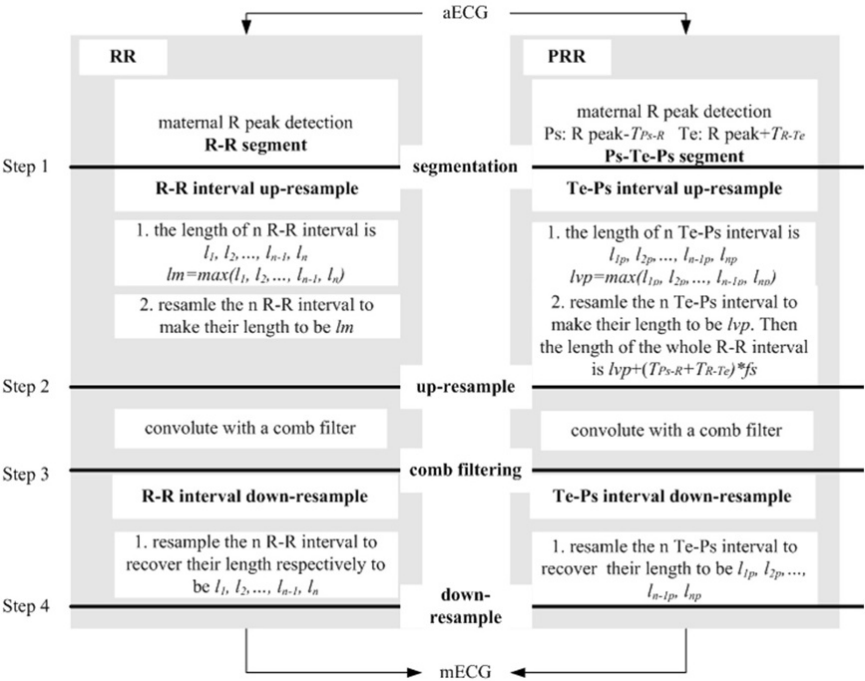
**Core algorithm block diagram for RR and PRR.**

According to the conclusion earlier mentioned, combining the advantages of LP and RR method, a modified fECG extraction algorithm is proposed. The core algorithm is shown in Figure 5. Unlike RR method, the proposed algorithm only resamples part of each R-R interval, so it is called Partially R-R Resampling based comb filter i.e. PRR.

The proposed core steps of PRR are further described as follows:

Step 1 in Figure 5: segmentation. Detect the maternal R peaks in aECG. Take the Ps point as the point *T*_*Ps-R*_ s before each R peak and Te point as the point *T*_*R-Te*_ s after each R peak. Then a complete cardiac cycle can be divided as Ps-Te-Ps. Ps-Te period is the previously mentioned heart beat span and Te-Ps period is the diastolic period.

Step 2 in Figure 5: Te-Ps interval up-resample. Denote the lengths of diastolic period as *l*_*1p*_*, l*_*2p*_*,…, l*_*np-1*_*, l*_*np*_ (points). Each diastolic period is resampled to have the same number of samples *l*_*vp*_. *l*_*vp*_ can be the maximum one of *l*_*1p*_*, l*_*2p*_*,…, l*_*np-1*_*, l*_*np*_. Thus every diastolic period is up resampled. And every heart beat span keeps unchanged. The total length of a cardiac cycle after resampling is *L* = (*T*_*Ps-R*_ + *T*_*R-Te*_)*fs + *l*_*vp*_, here *fs* is the sampling rate .

Step 3 in Figure 5: comb filtering. The up-resampled periodic signal convolutes with a comb filter. The designing detail for a comb filter can be found in literature [16]. The result is an estimation of up-resampled mECG.Step 4 in Figure 5: Te-Ps interval down-resample. Resample diastolic periods of the estimated up-resampled mECG to recover their original lengths. It turns out to be the estimation to mECG. The residual ECG (rECG) is obtained after subtracting it from the original aECG. The rECG can be seen as the estimation to fECG; of course, the noise can be weakened in a further step to get a clearer fECG which is not discussed in the paper.

In above Step 1, the *T*_*Ps-R*_ and *T*_*R-Te*_ values have slight difference for different individuals. *T*_*Ps-R*_ lies between 0.15 and 0.20 s, while *T*_*R-Te*_ lies 0.20 to 0.35 s [29]. In reality, it is difficult to detect the points Ps and Te steadily online, since P wave and T wave may be invisible in aECG. Therefore, we set *T*_*Ps-R*_ =0.2 s and *T*_*R-Te*_ = 0.4 s fixedly in the proposed PRR. A further illustration about this can be found in the Discussion Section.

## Results

The performance of the PRR algorithm is tested on synthetic mECG and aECG data and real antenatal abdominal recordings. The RR and LP are also implemented for comparison. In LP, the template window is chosen as the window between *R peak -*5/12 *T* and *R peak* + 7/12 *T*, here *T* is the average length of several R-R intervals [17, 18].

### Tests on synthetic data

**Figure 6:**
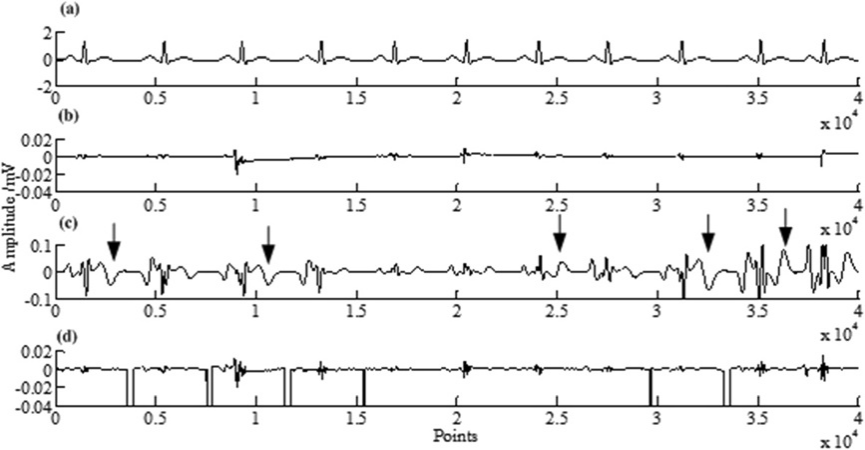
**Estimation errors for PRR, RR and LP when** ***r*** **= 0.4. (a)** the original synthetic ECG, **(b)** the error by PRR, **(c)** the error by RR, **(d)** the error by LP.

The simulated synthetic data is created as follows: (1) generate the ECG without HRV as in literature [33]; (2) change the length of each Te-Ps interval, to make it has HRV and constant heart beat span. The new length of a changed Te-Ps interval *N* = (1 + *r* × *rand*) × *n*, here n is the original length of a Te-Ps interval, *rand* is a random number between -1 and 1, and *r* is a set number between 0 and 1, called HRV variation coefficient. The bigger *r*, the larger signal’s HRV. Figure 6(a) gives an example of the synthetic ECG with *r* = 0.4.

For the simulated synthetic data showed in Figure 6(a), its ECG component is estimated by PRR, RR and LP respectively. The estimation errors are calculated and showed in Figure 6(b-d) respectively, in which *error* = eECG-ECG, with eECG representing the estimation of ECG. The root mean squares (RMS) of the estimation errors are also calculated, which are 0.0159, 0.0234 and 0.0419 respectively.

**Figure 7:**
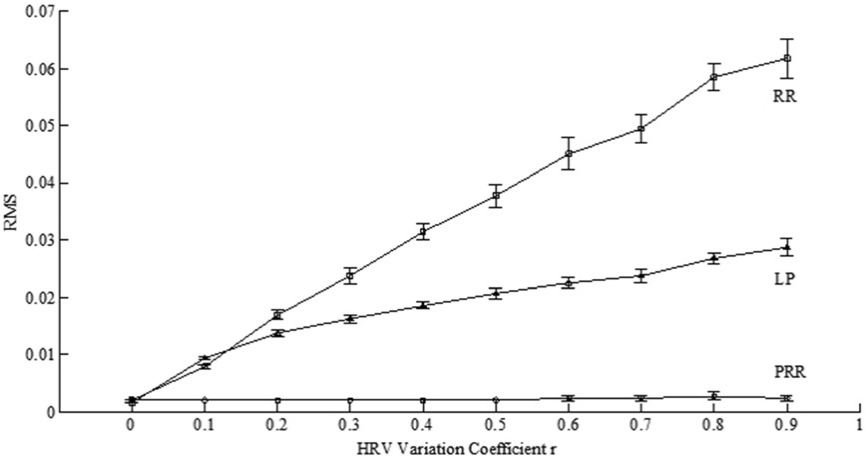
**RMSs of estimation errors for different HRV variation coefficient** ***r.***

Further take *r* as 0, 0.1, …, 0.8, 0.9 respectively to generate synthetic data to test. Create 20 synthetic ECG data for each *r*. The tested results are depicted with an errorbar plot as Figure 7, in which the Y-axis represents RMS value of the estimation errors.

We also generate 20 synthetic aECGs to test. For each synthetic aECG, we fixedly set *r* = 0.4 to create its mECG component and *r* = 0 to create its fECG component. In the test, the mECG components are estimated and subtracted from aECGs, resulting in rECGs. Then the added fECG components are excluded from rECGs, obtaining the resultant errors, whose *wave power ratios (WPR* s) are listed in Table 2. The *WPR* is defined as

**Table 2:** ***WPRs*** **using PRR, RR and LP for 20 synthetic aECGs**

No.	1	2	3	4	5	6	7	8	9	10
PRR × 10^-2^	0.403	0.857	0.504	1.376	0.514	2.324	0.581	1.768	1.873	0.442
RR × 10^-2^	2.507	2.274	2.075	2.272	1.523	3.978	1.939	3.199	3.428	2.521
LP × 10^-2^	0.447	0.920	0.570	1.616	0.557	2.556	0.667	1.910	2.249	0.494
No.	11	12	13	14	15	16	17	18	19	20
PRR × 10^-2^	0.687	0.480	0.541	0.784	0.500	0.520	0.531	0.604	0.455	1.072
RR × 10^-2^	2.029	1.867	2.252	1.770	2.405	1.989	1.801	2.935	2.114	1.445
LP × 10^-2^	0.762	0.540	0.591	0.909	0.517	0.619	0.650	0.674	0.505	1.135



here *s* and *e* are respectively the point 0.2 s before and 0.4 s after *i*-th R peak. *N* is the number of R peaks. The numerator measures the residual mECG component power in rECG, while the denominator mainly measures the original mECG power in aECG. The *WPR* indicates how much mECG component is kept in rECG. Figure 8 has illustrated estimation errors for a specific aECG when using three methods, and their *WPR* calculated are 0.0034, 0.0166 and 0.0048 respectively.

**Figure 8:**
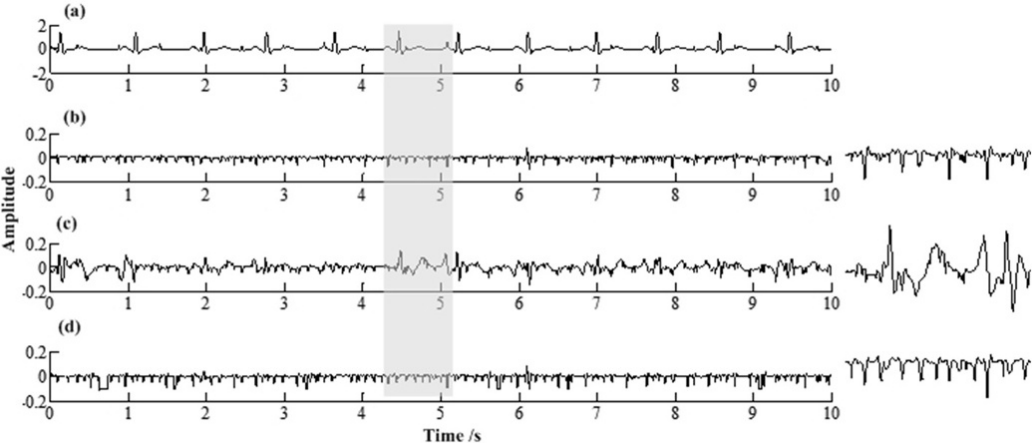
**Estimation errors for an aECG (mECG HRV** ***r*** **= 0.4). (a)** synthetic aECG, **(b)** estimation error by PRR, **(c)** estimation error by RR, and **(d)** estimation error by LP. Zoom of the grey segments are showed on the right.

From Figure 6(b-d) and Figure 8(b-d), it’s quite clear that: (1) RR method always has quite serious erroneous estimations on P and T waves; (2) LP method may have some transient errors at the edges of a diastolic period; (3) for PRR and LP, their estimations to the QRS complexes are almost the same. From Figure 7 it’s showed that: when *r* = 0, i.e. ECG has no HRV, the RMSs of errors for synthetic ECGs are the same for the three method; for others, RMSs for RR and LP increase with *r* but RMSs for PRR stay the same. Generally speaking, the tests on synthetic ECG show that PRR can estimate the ECG signal more accurately especially when it has large HRV.

### Experiments on real data

Tests are taken on data from MIT ADFED [30, 31] which contains five real-life records. Each record has five channels - one direct fECG and four abdominal ECG. The sampling rate is 1 KHz and their sampling durations are five minutes.

**Figure 9:**
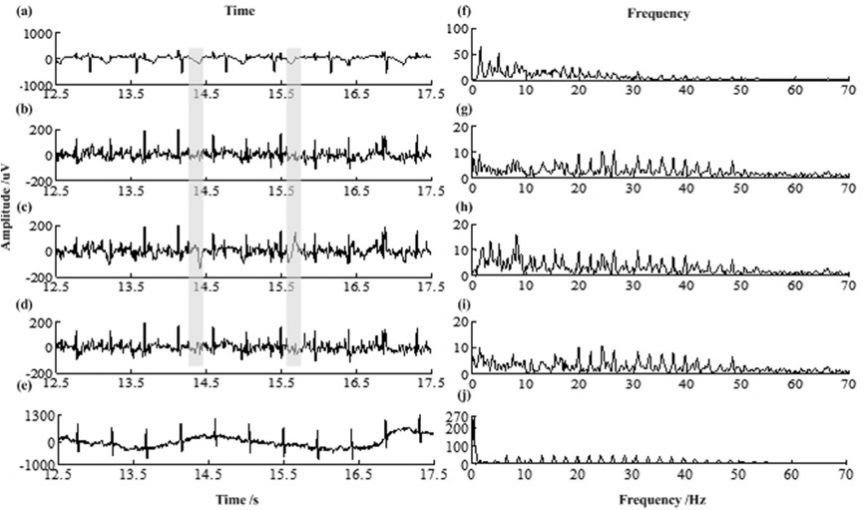
**The rECG for “r01” by the proposed method PRR, RR and LP. (a)** abdominal ECG, **(f)** spectrum of abdominal ECG, **(b)** residual ECG obtained by PRR, **(g)** spectrum of the residual ECG obtained by PRR, **(c)** residual ECG obtained by RR, **(h)** spectrum of the residual ECG obtained by RR, **(d)** residual ECG obtained by LP, **(i)** spectrum of the residual ECG obtained by LP, **(e)** the corresponding direct fECG and **(j)** spectrum of the direct fECG. (The grey segments indicate the residue of mECG complexes).

First, take record “r01” to test. 12 pieces of 5 second-length signals from 2.5 s to 62.5 s of the whole record are chosen. According to the steps described in Figure 5, mECGs are estimated and rECGs are obtained for these signals. rECGs and their spectrums by PRR, RR and LP respectively are depicted in Figure 9(b-e, g-j). From Figure 9, the three methods have almost same residual maternal QRS complexes; however, RR method has larger residual maternal T wave than the other two methods. The *WPRs* for rECGs of the 12 pieces are listed in Table 3. We can see that the *WPR*s for PRR are always the smallest among the three methods.

**Table 3:** ***WPR*** s for rECGs of record “r01” (*indicate the signal piece showed in Figure 9 **)**

No.	1	2	*3	4	5	6	7	8	9	10	11	12
PRR	**0.0723**	**0.0718**	**0.0739**	**0.0714**	**0.0702**	**0.0644**	**0.074**	**0.0631**	**0.0863**	**0.0750**	**0.0702**	**0.1056**
RR	0.0782	0.095	0.1098	0.0881	0.0777	0.0724	0.0756	0.0726	0.0884	0.0790	0.0747	0.1084
LP	0.088	0.0775	0.0747	0.0761	0.0834	0.0653	0.0856	0.0693	0.0945	0.0810	0.0728	0.1091

**Figure 10:**
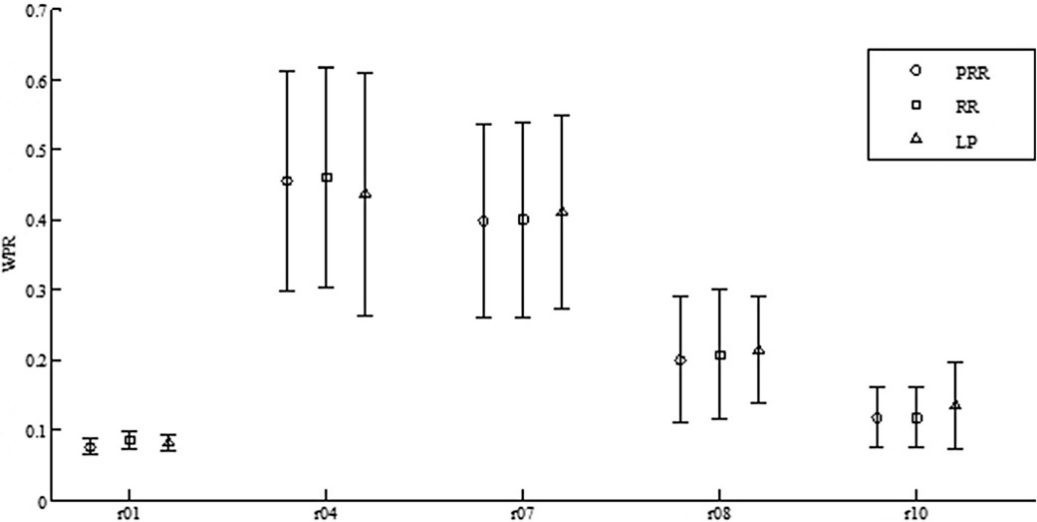
**The errorbar of** ***WPRs*** **for five real records.**

The errorbar of *WPRs* for all the five records are depicted in Figure 10. Generally speaking, the *WPR*s for PRR are the smallest.

## Discussion

### On invariant heart beat span

Based on the lengths of ECG waves for recordings in Set A and Set B, we get a conclusion that for normal sinus rhythm when the instant heart beat interval changes the heart beat span keeps almost unchanged, while the length of diastolic period fluctuates with heart beat interval.

HRV is always explained by the autonomic nerve’s regulation of heart and circulation system [34]. The activity of parasympathetic or sympathetic nervous system affects the sinus node’s beating rhythm [26, 27], and further affect the heart beat to have HRV. However, autonomic nerve may affect HRV just by determining the beat beginning point, i.e., Ps point on ECG, don't control the duration of heart being excited, i.e., heart beat span. The heart beat span for a person should only be determined by his or her own heart's physiology and healthy condition itself. Therefore, if there is no conduction block disease, the HRV for normal sinus rhythm just affects the variation of the length of Te-Ps interval*.* The lengths of P wave, P-R interval, QRS wave and S-T interval are independent of the heart beat rate [26], which constituting the stable heart beat span.

### Individual difference’s effect on PRR performance

In Method Section we set *T*_*Ps-R*_ and *T*_*R-Te*_ to be fixed. But actually different individuals may have slight difference on them. Now we discuss the effect below.

**Figure 11:**
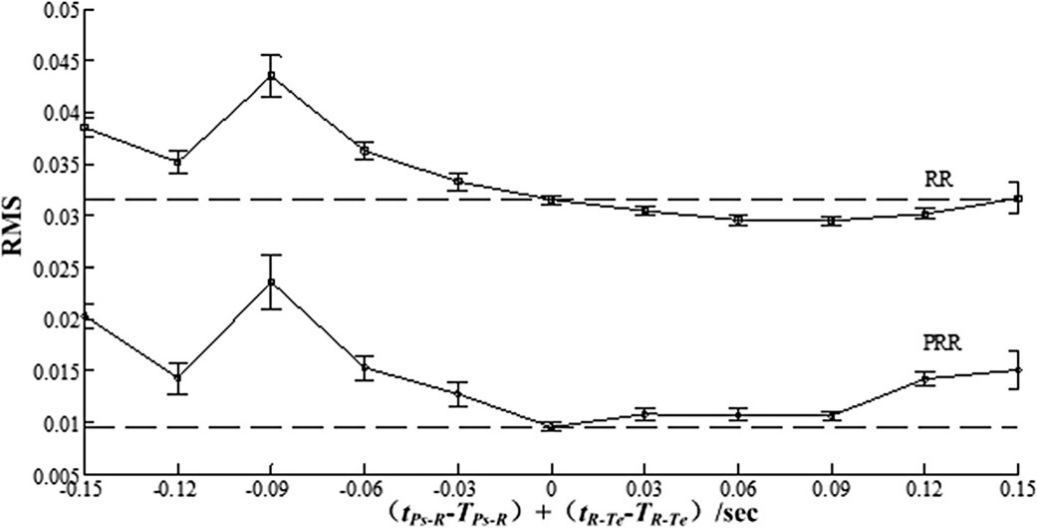
**The RMS for synthetic signals with different** ***t***_***Ps-R***_ **and** ***t***_***R-Te***_ **.**

Generating 11 synthetic ECG signals as Result Section described. Their *r* is 0.4. The real lengths of Ps-R *t*_*Ps-R*_ and R-Te *t*_*R-Te*_ intervals for these signals and their offsets to *T*_*Ps-R*_ and *T*_*R-Te*_ are listed in Table 4. The RMS for estimation errors by using PRR and RR are given in Figure 11. For PRR when *T*_*Ps-R*_ and *T*_*R-Te*_ get closer to their real values, the RMS becomes smaller; when they are equal the RMS-curve gets its minimum. The offset between them indeed affects PRR’s performance. In addition, no matter how large the offset is, the proposed method outperforms RR method. Considering the difficulty in P wave detection and its lower amplitude compared with T wave, in PRR *T*_*Ps-R*_ can be set as a fixed value while *T*_*R-Te*_ can be decided online according to the T peak detection.

**Table 4:** **The real lengths of Ps-R and R-Te intervals and their offsets to the fixed values for the 11 synthetic signals**

***t***_***Ps-R***_ (s)	0.15	0.16	0.17	0.18	0.19	0.2	0.21	0.22	0.23	0.24	0.25
*t*_*R-Te*_ (s)	0.30	0.32	0.34	0.36	0.38	0.4	0.42	0.44	0.46	0.48	0.50
(*t*_*Ps-R*_-*T*_*Ps-R*_) + (*t*_*R-Te*_-*T*_*R-Te*_)	-0.15	-0.12	-0.09	-0.06	-0.03	0	0.03	0.06	0.09	0.12	0.15

## Conclusions

With statistical analysis to the lengths of ECG waves from two databases, the conclusion comes out: heart beat span is invariant while the heart beat interval changes all the time. According to this prior information, a new modified fECG extraction algorithm is proposed called PRR. It copes with the mECG estimation inaccuracy caused by HRV and enhances the signal to noise ratio of the remaining fECG signal. Experiments show that the larger the HRV, the more obvious the advantage of PRR over other methods. So the proposed PRR method is outstanding on robustness, and it is believed it can be used into the practical fECG extraction. The computation complexity of this method can be studied in further work.
